# Pre-Procedural Anxiety and Associated Factors Among Women Seeking for Cervical Cancer Screening Services in Shenzhen, China: Does Past Screening Experience Matter?

**DOI:** 10.3389/fonc.2022.857138

**Published:** 2022-07-06

**Authors:** Wei Lin, Weikang Huang, Chaofan Mei, Chuyan Zhong, Leilei Zhu, Peiyi Liu, Shixin Yuan, Zhihua Liu, Yueyun Wang

**Affiliations:** ^1^ Department of Healthcare, Affiliated Shenzhen Maternity and Child Healthcare Hospital, Southern Medical University, Shenzhen, China; ^2^ Research Team of Cervical Cancer Prevention Project in Shenzhen, Affiliated Shenzhen Maternity and Child Healthcare Hospital, Southern Medical University, Shenzhen, China; ^3^ Department of Gynecology, Affiliated Shenzhen Maternity and Child Healthcare Hospital, Southern Medical University, Shenzhen, China; ^4^ Research Institute of Maternity and Child Healthcare, Affiliated Shenzhen Maternity and Child Healthcare Hospital, Southern Medical University, Shenzhen, China

**Keywords:** cervical cancer, past screening experience, pre-procedural anxiety, psychological harm, associated factor

## Abstract

**Background:**

Research gaps exist in addressing the psychological harm related to the cervical cancer screening. Anxiety is the most common distress driven by the screening procedures, which may be affected by past screening experience (PSE) but with uncertainty. This study aimed to evaluate the pre-procedural anxiety in cervical cancer screening and to identify the influence attributed to PSE.

**Methods:**

A cross-sectional survey targeted women seeking for cervical cancer screening services was conducted from June 5th to December 31st, 2020 in Shenzhen. The 20-item state anxiety scale of the State-Trait Anxiety Inventory (STAI-S) was applied to measure pre-procedural anxiety, in which a score of 40 or higher was regarded with anxiety symptom. Logistic regression models were established to explore potential associated factors of pre-procedural anxiety both for women with and without PSE.

**Results:**

Overall, 3,651 women were enrolled, in which 36.1% had never been screened and the remaining 63.9% had been screened at least once before. Women without PSE demonstrated more prevalent pre-procedural anxiety (74.5% vs. 67.8%, P <0.001) than their experienced counterparts. Among women without PSE, having heard of cervical cancer screening was associated with a lower likelihood of pre-procedural anxiety (OR: 0.37, 95%CI: 0.25~0.56). Among experienced women, participating three or more times screening was negatively associated with anxiety symptom (OR: 0.67, 95%CI: 0.53~0.84), however, both receiving screening within three years (OR: 1.58, 95%CI: 1.27~1.97) and unknowing previous screening results (OR: 1.42, 95%CI: 1.11~1.82) increased the susceptibility of pre-procedural anxiety.

**Conclusions:**

Women participating in cervical cancer screening commonly present pre-procedural anxiety. The association between PSE and pre-procedural anxiety may be influenced by past screening times, interval, and results. Psychological counseling according to women’s PSE before cervical cancer screening is warranted of necessity.

## Introduction

Cervical cancer is the fourth frequent malignancy in females worldwide (1). Almost all cancers in the cervix were caused by the high-risk human papillomavirus (HPV) (2). Routine cervical screening is one of the most essential prevention strategies, leading to great success in reducing the disease burden. However, women are required to receive gynecological procedures for cervical examination and sampling, which can be regarded as an invasive operation performed by a healthcare provider. Screening related procedures may act as stressors and bring adverse psychological outcomes. Notably, evidence about the psychological harm of cervical screening is restricted to distress induced by switching screening methods, receiving abnormal results, and following colposcopy related procedures (3–5). Research gaps exist in addressing the psychological harm before and during the screening process.

Recent systematic reviews identified the psychological harm of cancer screening procedures, in which anxiety was the most commonly assessed construct (6, 7). Anxiety is thought to be a future-oriented affective status that reflects one’s preparation to cope with uncertainty but possibly negative situations without a triggering stimulus (8). Anxious feelings may be prevalent when women treat pain as the most important determinant of cervical screening participation (9). However, scant studies examined cervical screening related anxiety and only followed non-mainstream screening methods, like optical spectroscopy and visual inspection (10, 11). Anxiety driven by HPV or cytology based methods remain to be investigated.

Past screening experience (PSE) may impact on cervical cancer screening related anxiety. Anxious feelings could appear among those without PSE due to uncertainty of screening procedures. For women who have ever been screened, on one hand, they may prefer less frequent screening in order to avoid frequent anxiety, worry, or nervousness (12). On the other hand, anxiety may also be alleviated by repeated participation and fully understanding of screening procedures, as exposure to the feared situation helps to deal with specific anxiety (13). As more and more females are encouraged to receive cervical screening, there is a urgent need to understand screening related anxiety among women with and without PSE. Hence, based on a cross-sectional survey in Shenzhen, we evaluated the pre-procedural anxiety and associated factors among women seeking for cervical cancer screening services, in order to address the dearth of information about the psychological harm associated to cervical cancer screening and to identify the influence attributed to PSE.

## Materials and Methods

### Study Setting and Participants

A cross-sectional survey has been conducted from June 5^th^ to December 31^st^, 2020 in Pinghu Maternity-child Healthcare and Family Planning Service Center of Longgang District, Shenzhen. It has been one of the most influential and public screening centers funded by the local government, offering free screening services of common diseases for nearly 8,000 women per year. Women could have access to cervical cancer screening services if they were engaged in sexual behavior, not pregnant, and at an age range from 20 to 65 years old. During the survey period, women who came to this screening site seeking for cervical cancer screening services and met above criteria were invited to participate in our survey. Here, we exclueded women without a smartphone or incapacitated women due to intellectual or other disability. They would be provided with a full explanation and invitation of the present survey by trained research assistants. With informed consent, women were asked to finish an online questionnaire before they received gynecological assessment and subsequent screening procedures. The questionnaire was available to access *via* scanning a unique quick response code with their smartphones, which was hosted by WenJuanXing (Changsha Haoxing Information Technology Co., Ltd., China). Totally, we collected 3717 questionnaires and excluded 66 questionnaires with unknown age information or out-of age range. Ethical approval was obtained from the medical ethics committee of Shenzhen Maternity and Child Healthcare Hospital.

### Measurement

#### Demographic Characteristics and Reproductive Health Condition

A structured questionnaire containing different aspects was employed in this study. Demographic characteristics were firstly collected based on self-report, containing age, ethnicity, local household registration, marital status, education level, occupation types, and monthly income level. Information on women’s reproductive health was also required, such as age at menarche and first sexual intercourse (coitarche), the number of sexual partners in recent one year, condom and oral contraceptive use, parity, age at first delivery, malignancy diagnosis of first-degree relatives, and previous diagnosis of vaginitis. Detailed division of above-mentioned variables were listed in **Table 1**.

**Table 1:** Characteristics of the participants varied by PSE (N=3651).

Variables	PSE		P value	Overall, n (%)
	Without, n (%)	With, n (%)		
Demographic characteristic				
Age (year)				
<41	765 (58.0)	1147 (49.2)	<0.001	1912 (52.4)
≥41	554 (42.0)	1185 (50.8)		1739 (47.6)
Ethnicity				
Han	1211 (91.8)	2197 (94.2)	0.005	3408 (93.3)
Others	108 (8.2)	135 (5.8)		243 (6.7)
Local household registration				
Yes	184 (13.9)	503 (21.6)	<0.001	687 (18.8)
No	1135 (86.1)	1829 (78.4)		2964 (81.2)
Marital status				
Single/divorced/widow	87 (6.6)	105 (4.5)	0.006	192 (5.3)
Married	1232 (93.4)	2227 (95.5)		3459 (94.7)
Education level				
Junior middle school or below	859 (65.1)	1321 (56.6)	<0.001	2180 (59.7)
Senior middle school	254 (19.3)	575 (24.7)		829 (22.7)
College or above	206 (15.5)	436 (18.7)		642 (17.6)
Occupation types				
Administrator/professional	139 (10.5)	266 (11.4)	0.017	405 (11.1)
Worker	520 (39.4)	787 (33.7)		1307 (35.8)
Business services personnel	178 (13.5)	343 (14.7)		521 (14.3)
Housewife/unemployed woman	332 (25.2)	632 (27.1)		964 (26.4)
Others	150 (11.4)	304 (13.0)		454 (12.4)
Monthly income (RMB)				
<5,000	1030 (78.1)	1758 (75.4)	0.065	2788 (76.4)
≥5,000	289 (21.9)	574 (24.6)		863 (23.6)
Reproductive health condition				
Age at menarche (year)				
<12	32 (2.4)	71 (3.0)	0.052	103 (2.8)
12 to 15	1053 (79.8)	1781 (76.4)		2834 (77.6)
≥16	234 (17.7)	480 (20.6)		714 (19.6)
Age at coitarche (year)				
<18	119 (9.0)	130 (5.6)	<0.001	249 (6.8)
18 to 24	954 (72.3)	1698 (72.8)		2652 (72.6)
≥25	246 (18.7)	504 (21.6)		750 (20.5)
The number of sexual partners in recent one year				
0	116 (8.8)	157 (6.7)	0.020	273 (7.5)
1	1113 (84.4)	2044 (87.7)		3157 (86.5)
≥2	90 (6.8)	131 (5.6)		221 (6.1)
Consistent condom use during sexual intercourse				
No	1117 (84.7)	1954 (83.8)	0.48	3071 (84.1)
Yes	202 (15.3)	378 (16.2)		580 (15.9)
Oral contraceptive use				
Never	1094 (82.9)	1947 (83.5)	0.67	3041 (83.3)
Ever	225 (17.1)	385 (16.5)		610 (16.7)
Parity				
0	52 (3.9)	43 (1.8)	<0.001	95 (2.6)
1	334 (25.3)	587 (25.2)		921 (25.2)
2	660 (50.0)	1258 (53.9)		1918 (52.5)
≥3	273 (20.7)	444 (19.0)		717 (19.6)
Age at first delivery (year)^a^				
<18	160 (12.6)	287 (12.5)	0.099	447 (12.2)
18 to 24	647 (51.1)	1095 (47.8)		1742 (47.7)
25 to 29	371 (29.3)	761 (33.2)		1132 (31.0)
≥30	89 (7.0)	146 (6.4)		235 (6.4)
Malignancy diagnosis of first-degree relatives				
No/unknown	1251 (94.8)	2143 (91.9)	0.001	3394 (93.0)
Yes	68 (5.2)	189 (8.1)		257 (7.0)
Previous diagnosis of vaginitis				
No	928 (70.4)	1334 (57.2)	<0.001	2262 (62.0)
Yes	391 (29.6)	998 (42.8)		1389 (38.0)
Health habit				
Active smoking				
Never	1282 (97.2)	2300 (98.6)	0.002	3582 (98.1)
Ever	37 (2.8)	32 (1.4)		69 (1.9)
Passive smoking				
Never	1131 (85.7)	1967 (84.3)	0.26	3098 (84.9)
Ever	188 (14.3)	365 (15.7)		553 (15.1)
Sitting hours per day				
<5	732 (55.5)	1337 (57.3)	0.28	2069 (56.7)
≥5	587 (44.5)	995 (42.7)		1582 (43.3)
Walking steps per day				
<5000	855 (64.8)	1353 (58.0)	<0.001	2208 (60.5)
≥5000	464 (35.2)	979 (42.0)		1443 (39.5)
Frequency of physical exercise per week				
0	610 (46.2)	735 (31.5)	<0.001	1345 (36.8)
1	367 (27.8)	684 (29.3)		1051 (28.8)
2	206 (15.6)	495 (21.2)		701 (19.2)
≥3	136 (10.3)	418 (17.9)		554 (15.2)
Psychological health status				
Psychological distress in recent two weeks				
No	1074 (81.4)	1849 (79.3)	0.12	2923 (80.1)
Yes	245 (18.6)	483 (20.7)		728 (19.9)

a Nonparous women were not included. Bold values indicate statistical significance (P < 0.05).

#### Past Cervical Screening Experience

All women were asked to recall previous experience of cervical cancer screening. The past participation of screening was evaluated by asking “*Before the survey time, approximately how many times have you ever participated in cervical cancer screening? (none/once/twice/three or more times)*”. Women without PSE were assessed with the awareness of cervical cancer screening service *via* asking “*Before the survey time, have you ever heard of cervical cancer screening? (yes/no)*”. Specific questions was developed to query past screening experience, containing “*When did you receive previous screening? (within/over 3 years)*” and “*What was the result of previous screening? (normal/abnormal/unknown)*”.

#### Health Habit

We further gathered variables of health habits in their daily routines. Women needed to recall specific life events, including active and passive exposure to smoking, the duration of sitting per day, the number of walking steps per day, and the frequency of physical exercise per week. Here, active smoking was defined as ever or currently smoking at least one cigarette per day on average. In addition, women exposed to tobacco smoke more than 15 minutes, at least one day per week were regarded with passive smoking. Walking steps were calculated according to the pedometer function of their smart-phones. Physical exercise referred to common exercise forms, including sports, running, swimming, dancing, mountain climbing, rope skipping, etc.

#### Psychological Health Status

Recent psychological health of the participants was measured through an ultra-brief screening scale named the Patient Health Questionnaire-4 (PHQ-4). It consists of a 2-item anxiety scale and a 2-item depression scale, assessing the frequency of psychological distress in recent two weeks. Each item was rated in four response options (not at all=0, several days=1, more than half of the days=2, and almost every day=3). Therefore, a total score of the four items was ranged from 0 to 12. Suggested by previous validation (14), those who scored ≥3 on PHQ-4 were considered to have psychological distress. In the present study, the internal consistency reliability of the PHQ-4 was found to be acceptable (Cronbach’s α: 0.86).

#### Pre-Procedural Anxiety

Pre-procedural anxiety was assessed by the state anxiety scale of the State-Trait Anxiety Inventory (STAI-S). The STAI-S is composed of 20 items that reflect the transient emotional response to a stressful situation. It measures the anxious symptom at the moment of scoring, which has been widely adopted to identify anxiety in the Chinese population (15). Hence, the participants in our survey were all required to finish the STAI-S prior to gynecological procedures, in order to figure out their present feelings. All items of the STAI-S were responded on a 4-point Likert-type scale, contributing to a total score of 20 to 80. The score of the STAI-S positively correlates with the severity of anxiety. A total score of 40 or higher was applied to reflect anxious symptom in the present study, in line with past investigations (15–17). The Cronbach’s α of STAI-S in this study was 0.88.

### Statistical Analyses

All data were analyzed descriptively by means of the SPSS 21.0 software (IBM Corp., Armonk, NY). Categorical variables were presented with numbers and frequencies, and continuous data were presented with means and standard deviations. For women with different characteristics (demographics, reproductive health condition, health habits, etc), the chi-square test was applied to detect the difference of anxiety level across subgroups, while the t-test and one way ANOVA were used to compare the distributed difference of STAI-S score. Logistic regression models were established to explore potential associated factors of pre-procedural anxiety both for women with and without PSE. Variables with P ≤0.10 in the uni-variate analysis were included in the multi-variate logistic regression models. Associated factors were identified with the stepwise procedure. Odds ratios (OR) and 95% confident intervals (CI) were calculated to estimate the strength of associations. Statistical significance was set to be less than 0.05 with a two-tailed test.

## Results

### Characteristics of All Participants

**Figure 1:**
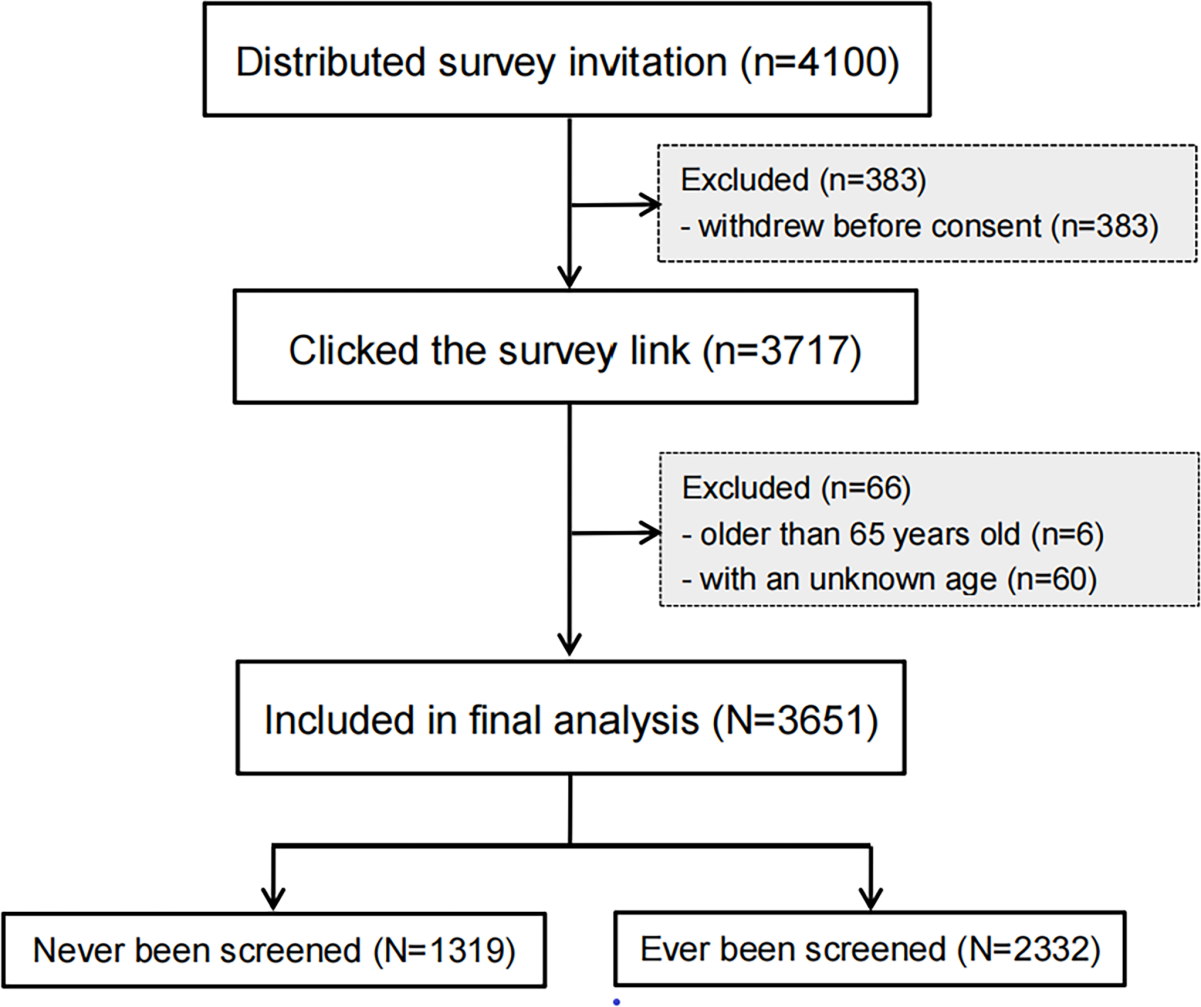
Flow chart diagram of the study population.

**Figure 2:**
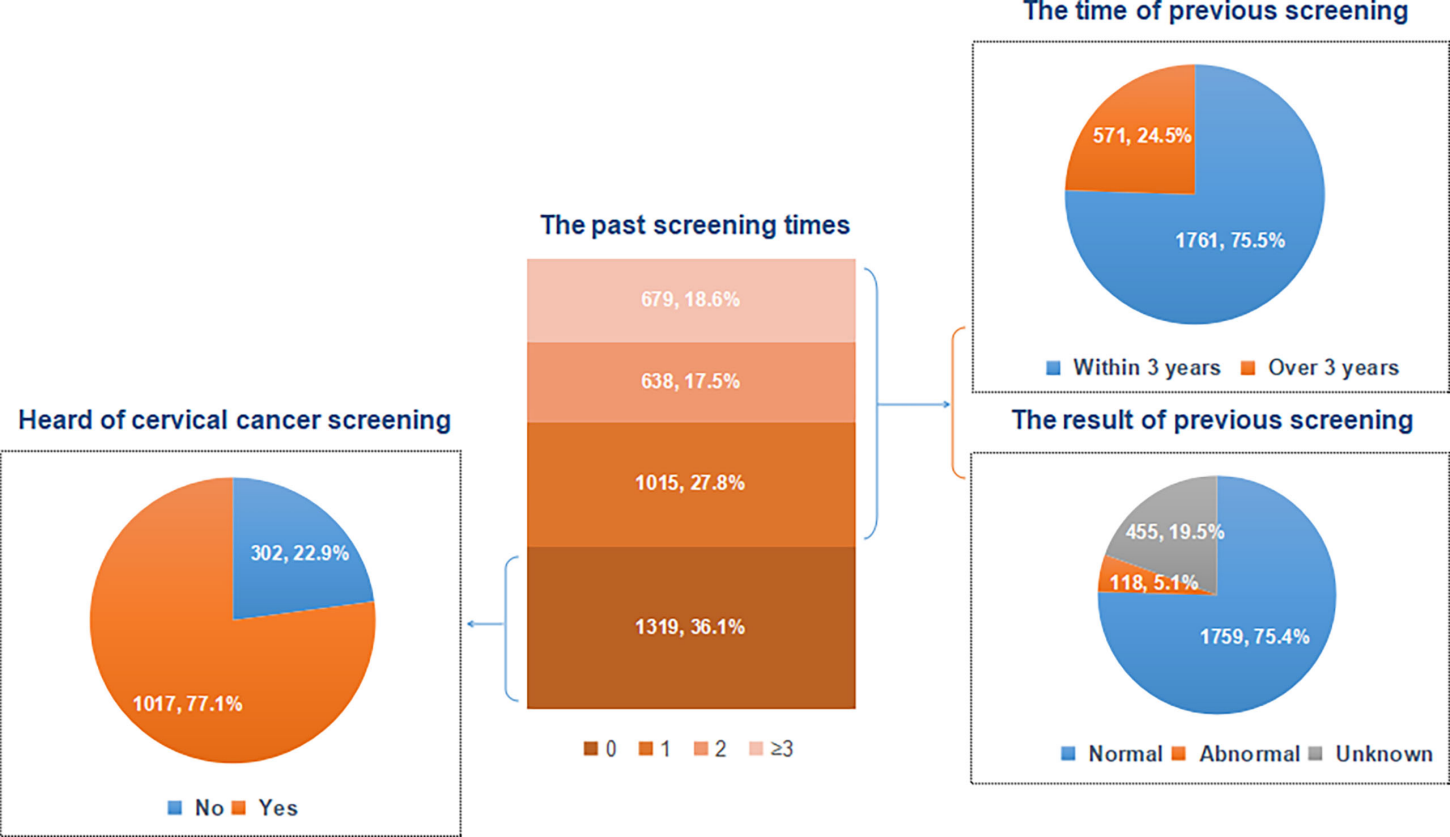
Past cervical cancer screening experience of all participants.

In total, 3,651 women were included in analysis (**Figure 1**), with an average age of 40.65 years (standard deviation: 7.56). Of all participants, 36.1% had never been screened before, while the remaining women had been screened at least once (once: 27.8%, twice: 17.5%, and three times or more: 18.6%) (**Figure 2**). Moreover, 302 women never heard of cervical cancer screening, accounted for 22.9% of non-experienced women. Among women with PSE, approximately three quarters received screening services within recent three years and reported normal screening results.

Characteristics of the participants varied by PSE (**Table 1**). Compared to those without PSE, experienced women were likely to be older, Han ethnic, local household registered, married, and well-educated (all P <0.05). These two groups also varied in occupation types, age at coitarche, the number of sexual partners, parity, malignancy diagnosis of first-degree relatives, and previous diagnosis of vaginitis (all P <0.05). Furthermore, experienced women tended to have healthier habits, such as no smoking, walking more steps, and more frequent physical exercise (all P <0.05).

### Prevalence of Pre-Procedural Anxiety in Cervical Cancer Screening

**Figure 3:**
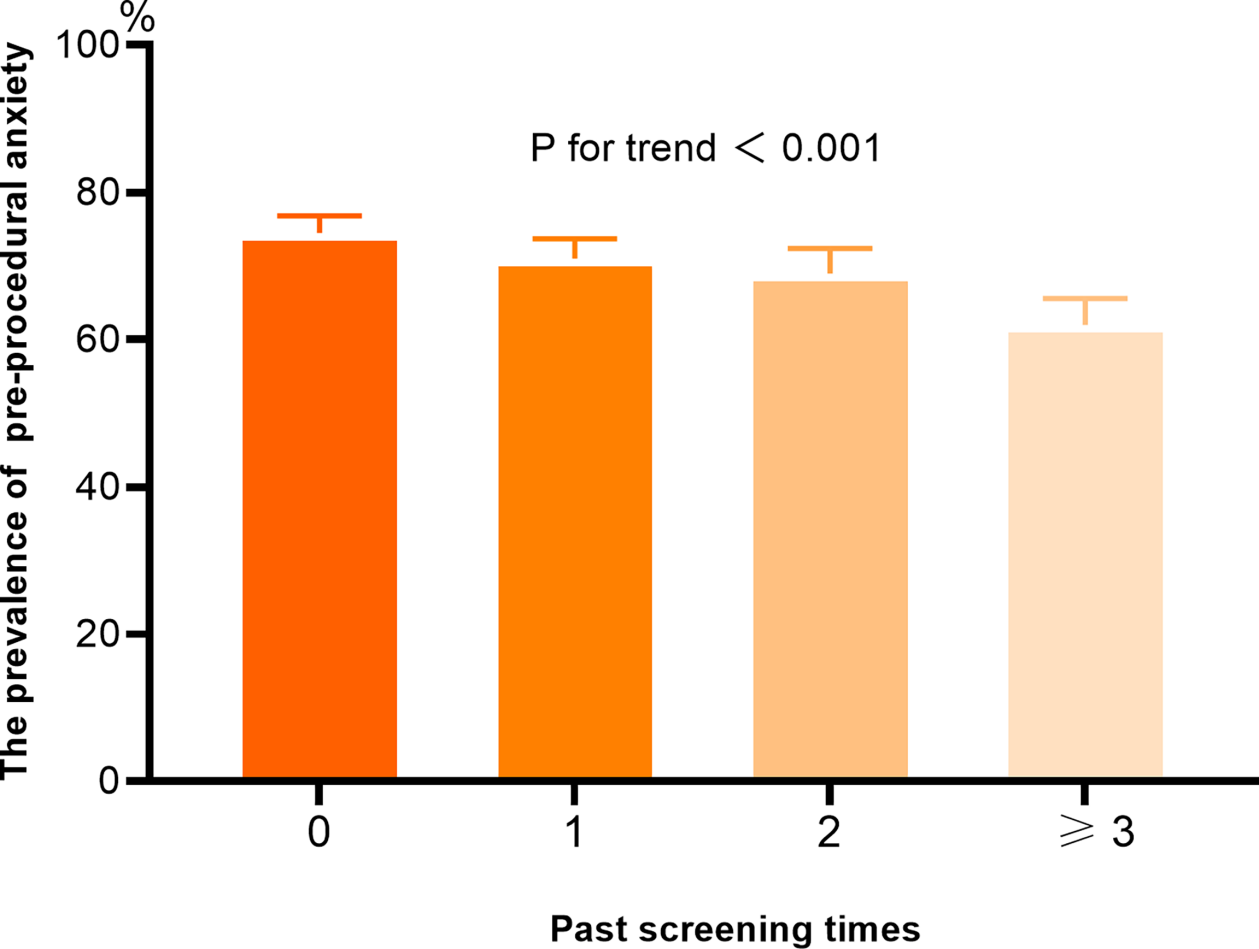
The prevalence of pre-procedural anxiety according to the past screening times.

The average score of STAI-S was 42.72 (standard deviation: 8.64) in this survey. Women without PSE demonstrated significantly higher score of STAI-S than those with PSE (43.64 vs. 42.28, P <0.001). When using a cut-off value of 40, the overall prevalence of pre-procedural anxiety was 70.3%. Compared to the experienced counterparts, a higher prevalence of anxious symptom was reported among women without PSE (74.5% vs. 67.8%, P <0.001). The prevalence of anxious symptom decreased with the increased times of past screening participation (P for trend <0.001) (**Figure 3**). Regardless of whether women had been screened before, distinct STAI-S scores and prevalence of anxiety were detected across subgroups of varied characteristics (**Tables 2**, **3**).

**Table 2:** Pre-procedural anxiety and associated factors among women without PSE (N=1319).

Variable	STAI-S score (mean, SD)	P value^a^	The prevalence of anxiety (n, %)	P value^b^	Uni-variate OR (95%CI)^c^	Multi-variate OR (95%CI)
Demographic characteristic						
Age (year)						
<41	42.47 (9.00)	<0.001	526 (68.8)	<0.001	1.00 (reference)	1.00 (reference)
≥41	45.25 (7.33)		457 (82.5)		2.14 (1.64, 2.80)	1.57 (1.15, 2.15)
Ethnicity						
Han	43.56 (8.42)	0.26	898 (74.2)	0.30	1.00 (reference)	
Others	44.55 (8.76)		85 (78.7)		1.29 (0.80, 2.08)	
Local household registration						
Yes	41.86 (9.94)	0.008	118 (64.1)	<0.001	1.00 (reference)	
No	43.93 (8.15)		865 (76.2)		1.79 (1.29, 2.49)	
Marital status						
Single/divorced/widow	42.51 (8.58)	0.20	55 (63.2)	0.012	1.00 (reference)	1.00 (reference)
Married	43.72 (8.44)		928 (75.3)		1.78 (1.13, 2.80)	2.33 (1.37, 3.97)
Education level						
Junior middle school or below	44.69 (7.70)	<0.001	686 (79.9)	<0.001	1.00 (reference)	1.00 (reference)
Senior middle school	42.86 (9.03)		178 (70.1)		0.59 (0.43, 0.81)	0.73 (0.52, 1.02)
College or above	40.20 (9.67)		119 (57.8)		0.35 (0.25, 0.48)	0.45 (0.31, 0.65)
Occupation types						
Administrator/professional	41.33 (9.85)	0.002	88 (63.3)	0.011	1.00 (reference)	
Worker	44.23 (7.40)		401 (77.1)		1.95 (1.31, 2.92)	
Business services personnel	43.47 (9.07)		131 (73.6)		1.62 (1.00, 2.61)	
Housewife/unemployed woman	44.24 (8.69)		256 (77.1)		1.95 (1.27, 3.00)	
Others	42.58 (8.79)		107 (71.3)		1.44 (0.88, 2.36)	
Monthly income (RMB)						
<5,000	44.08 (8.11)	0.001	793 (77.0)	<0.001	1.00 (reference)	
≥5,000	42.07 (9.42)		190 (65.7)		0.57 (0.43, 0.76)	
Reproductive health condition						
Age at menarche (year)						
<12	42.75 (8.56)	0.83	23 (71.9)	0.80	0.89 (0.41, 1.94)	
12 to 15	43.67 (8.54)		782 (74.3)		1.00 (reference)	
≥16	43.61 (8.05)		178 (76.1)		1.10 (0.79, 1.53)	
Age at coitarche (year)						
<18	45.57 (8.24)	0.004	97 (81.5)	0.041	1.00 (reference)	
18 to 24	43.69 (8.33)		715 (74.9)		0.68 (0.42, 1.10)	
≥25	42.49 (8.84)		171 (69.5)		0.52 (0.30, 0.88)	
The number of sexual partners in recent one year						
0	43.97 (8.18)	0.021	86 (74.1)	0.22	1.00 (reference)	
1	43.42 (8.59)		823 (73.9)		0.99 (0.64, 1.53)	
≥2	45.96 (6.63)		74 (82.2)		1.61 (0.82, 3.19)	
Consistent condom use during sexual intercourse						
No	43.80 (8.22)	0.14	844 (75.6)	0.043	1.00 (reference)	
Yes	42.74 (9.62)		139 (68.8)		0.71 (0.51, 0.99)	
Oral contraceptive use						
Never	43.91 (8.28)	0.017	832 (76.1)	0.005	1.00 (reference)	
Ever	42.33 (9.16)		151 (67.1)		0.64 (0.47, 0.88)	
Parity						
0	44.87 (8.46)	0.028	41 (78.8)	0.004	1.78 (0.88, 3.60)	
1	42.48 (8.60)		226 (67.7)		1.00 (reference)	
2	43.90 (8.54)		498 (75.5)		1.47 (1.10, 1.96)	
≥3	44.18 (7.95)		218 (79.9)		1.89 (1.30, 2.75)	
Age at first delivery (year)						
Nonparous	44.87 (8.46)	0.010	41 (78.8)	0.006	1.37 (0.65, 2.91)	2.38 (1.01, 5.61)
<18	44.04 (8.02)		501 (77.4)		1.26 (0.85, 1.87)	0.93 (0.61, 1.41)
18 to 24	43.37 (8.56)		117 (73.1)		1.00 (reference)	1.00 (reference)
25 to 29	43.58 (8.70)		271 (73.0)		1.00 (0.66, 1.51)	0.83 (0.53, 1.30)
≥30	40.74 (9.75)		53 (59.6)		0.54 (0.31, 0.94)	0.50 (0.28, 0.91)
Caner history of first-degree relatives						
No/unknown	43.77 (8.39)	0.013	938 (75.0)	0.11	1.00 (reference)	
Yes	41.16 (9.18)		45 (66.2)		0.65 (0.39, 1.10)	
Previous diagnosis of vaginitis						
No	44.04 (8.42)	0.008	706 (76.1)	0.046	1.00 (reference)	
Yes	42.69 (8.46)		277 (70.8)		0.76 (0.59, 1.00)	
Health habit						
Active smoking						
Never	43.63 (8.47)	0.88	956 (74.6)	0.83	1.00 (reference)	
Ever	43.84 (8.07)		27 (73.0)		0.92 (0.44, 1.92)	
Passive smoking						
Never	43.78 (8.38)	0.15	853 (75.4)	0.068	1.00 (reference)	
Ever	42.81 (8.88)		130 (69.1)		0.73 (0.52, 1.02)	
Sitting hours per day						
<5	43.93 (8.60)	0.16	561 (76.6)	0.049	1.00 (reference)	
≥5	43.27 (8.25)		422 (71.9)		0.78 (0.61, 1.00)	
Walking steps per day						
<5000	44.38 (8.21)	<0.001	672 (78.6)	<0.001	1.00 (reference)	1.00 (reference)
≥5000	42.27 (8.73)		311 (67.0)		0.55 (0.43, 0.71)	0.63 (0.48, 0.82)
Frequency of physical exercise per week						
0	45.06 (7.69)	<0.001	491 (80.5)	<0.001	1.00 (reference)	
1	42.58 (9.25)		262 (71.4)		0.61 (0.45, 0.82)	
2	42.11 (8.64)		137 (66.5)		0.48 (0.34, 0.68)	
≥3	42.41 (8.24)		93 (68.4)		0.52 (0.35, 0.79)	
Psychological health status						
Psychological distress in recent two weeks						
No	43.08 (8.50)	<0.001	779 (72.5)	0.001	1.00 (reference)	1.00 (reference)
Yes	46.08 (7.78)		204 (83.3)		1.88 (1.31, 2.70)	2.85 (1.94, 4.19)
Past cervical cancer screening experience						
Heard of cervical cancer screening						
No	46.93 (6.36)	<0.001	268 (88.7)	<0.001	1.00 (reference)	1.00 (reference)
Yes	42.66 (8.75)		715 (70.3)		0.30 (0.21, 0.44)	0.37 (0.25, 0.56)

a P for t test or one-way ANOVA.

b P for chi-square test.

c Variables with P ≤0.10 in the uni-variate analysis were included in the multi-variate logistic regression model. Bold values indicate statistical significance (P < 0.05).

**Table 3:** Pre-procedural anxiety and associated factors among women with PSE (N=2332).

Variable	STAI-S score (mean, SD)	P value^a^	The prevalence of anxiety (n, %)	P value^b^	Uni-variate OR (95%CI)^c^	Multi-variate OR (95%CI)
Demographic characteristic						
Age (year)						
<41	41.84 (8.94)	0.018	754 (65.7)	0.033	1.00 (reference)	
≥41	42.70 (8.46)		828 (69.9)		1.21 (1.02, 1.44)	
Ethnicity						
Han	42.25 (8.73)	0.49	1489 (67.8)	0.79	1.00 (reference)	
Others	42.78 (8.37)		93 (68.9)		1.05 (0.72, 1.53)	
Local household registration						
Yes	40.72 (9.54)	<0.001	297 (59.0)	<0.001	1.00 (reference)	
No	42.71 (8.42)		1285 (70.3)		1.64 (1.34, 2.01)	
Marital status						
Single/divorced/widow	41.83 (8.79)	0.59	70 (66.7)	0.79	1.00 (reference)	
Married	42.30 (8.71)		1512 (67.9)		1.06 (0.70, 1.60)	
Education level						
Junior middle school or below	43.38 (8.06)	<0.001	975 (73.8)	<0.001	1.00 (reference)	1.00 (reference)
Senior middle school	41.35 (9.20)		353 (61.4)		0.56 (0.46, 0.70)	0.61 (0.49, 0.77)
College or above	40.16 (9.40)		254 (58.3)		0.50 (0.40, 0.62)	0.59 (0.45, 0.77)
Occupation types						
Administrator/professional	40.17 (9.81)	<0.001	152 (57.1)	<0.001	1.00 (reference)	
Worker	43.19 (7.97)		578 (73.4)		2.07 (1.55, 2.77)	
Business services personnel	41.38 (8.86)		221 (64.4)		1.36 (0.98, 1.89)	
Housewife/unemployed woman	42.40 (8.84)		425 (67.2)		1.54 (1.15, 2.07)	
Others	42.53 (8.74)		206 (67.8)		1.58 (1.12, 2.22)	
Monthly income (RMB)						
<5,000	42.81 (8.47)	<0.001	1242 (70.6)	<0.001	1.00 (reference)	1.00 (reference)
≥5,000	40.66 (9.24)		340 (59.2)		0.60 (0.50, 0.73)	0.79 (0.64, 0.92)
Reproductive health condition						
Age at menarche (year)						
<12	41.63 (8.81)	0.28	49 (69.0)	0.19	1.10 (0.66, 1.84)	
12 to 15	42.16 (8.76)		1191 (66.9)		1.00 (reference)	
≥16	42.81 (8.51)		342 (71.2)		1.23 (0.98, 1.53)	
Age at coitarche (year)						
<18	43.87 (8.49)	0.092	97 (74.6)	0.23	1.00 (reference)	
18 to 24	42.23 (8.73)		1147 (67.6)		0.71 (0.47, 1.07)	
≥25	42.04 (8.69)		338 (67.1)		0.69 (0.45, 1.07)	
The number of sexual partners in recent one year						
0	43.57 (8.43)	<0.001	116 (73.9)	<0.001	1.00 (reference)	1.00 (reference)
1	41.95 (8.78)		1354 (66.2)		0.69 (0.48, 1.00)	0.80 (0.54, 1.18)
≥2	45.89 (6.85)		112 (85.5)		2.08 (1.14, 3.81)	2.11 (1.13, 3.93)
Consistent condom use during sexual intercourse						
No	42.44 (8.53)	0.058	1341 (68.6)	0.063	1.00 (reference)	
Yes	41.44 (9.54)		241 (63.8)		0.80 (0.64, 1.01)	
Oral contraceptive use						
Never	42.25 (8.68)	0.71	1329 (68.3)	0.33	1.00 (reference)	
Ever	42.43 (8.87)		253 (65.7)		0.89 (0.71, 1.12)	
Parity						
0	41.37 (9.08)	<0.001	26 (60.5)	0.001	0.87 (0.46, 1.64)	
1	41.40 (8.84)		374 (63.7)		1.00 (reference)	
2	42.19 (8.73)		849 (67.5)		1.18 (0.96,1.45)	
≥3	43.79 (8.27)		333 (75.0)		1.71 (1.30, 2.25)	
Age at first delivery (year)						
Nonparous	41.37 (9.08)	0.65	26 (60.5)	0.62	0.75 (0.39, 1.44)	
<18	42.55 (8.73)		757 (69.1)		1.09 (0.83, 1.44)	
18 to 24	42.30 (8.27)		193 (67.2)		1.00 (reference)	
25 to 29	41.99 (8.87)		506 (66.5)		0.97 (0.72, 1.29)	
≥30	42.00 (8.50)		100 (68.5)		1.06 (0.69, 1.62)	
Caner history of first-degree relatives						
No/unknown	42.36 (8.66)	0.15	1463 (68.3)	0.13	1.00 (reference)	
Yes	41.41 (9.28)		119 (63.0)		0.79 (0.58, 1.08)	
Previous diagnosis of vaginitis						
No	42.76 (8.60)	0.004	933 (69.9)	0.012	1.00 (reference)	1.00 (reference)
Yes	41.67 (8.82)		649 (65.0)		0.80 (0.67, 0.95)	0.77 (0.64, 0.92)
Health habit						
Active smoking						
Never	42.27 (8.71)	0.73	1558 (67.7)	0.38	1.00 (reference)	
Ever	42.81 (9.25)		24 (75.0)		1.43 (0.64, 3.20)	
Passive smoking						
Never	42.30 (8.68)	0.80	1337 (68.0)	0.75	1.00 (reference)	
Ever	42.18 (8.87)		245 (67.1)		0.96 (0.76, 1.22)	
Sitting hours per day						
<5	41.92 (8.90)	0.019	879 (65.7)	0.012	1.00 (reference)	1.00 (reference)
≥5	42.77 (8.43)		703 (70.7)		1.25 (1.05, 1.50)	1.25 (1.03, 1.51)
Walking steps per day						
<5000	43.14 (8.61)	<0.001	970 (71.7)	<0.001	1.00 (reference)	
≥5000	41.10 (8.72)		612 (62.5)		0.66 (0.55, 0.79)	
Frequency of physical exercise per week						
0	44.01 (8.27)	<0.001	552 (75.1)	<0.001	1.00 (reference)	1.00 (reference)
1	42.84 (8.31)		483 (70.6)		0.80 (0.63, 1.01)	0.92 (0.72, 1.17)
2	40.86 (8.77)		301 (60.8)		0.51 (0.40, 0.66)	0.66 (0.51, 0.86)
≥3	40.01 (9.28)		246 (58.9)		0.47 (0.37, 0.61)	0.63 (0.48, 0.83)
Psychological health status						
Psychological distress in recent two weeks						
No	41.30 (8.67)	<0.001	1186 (64.1)	<0.001	1.00 (reference)	1.00 (reference)
Yes	46.05 (7.80)		396 (82.0)		2.55 (1.98, 3.27)	3.00 (2.30, 3.91)
Past cervical cancer screening experience						
The total times of screening participation						
1	43.08 (8.48)	<0.001	721 (71.0)	<0.001	1.00 (reference)	1.00 (reference)
2	42.12 (8.60)		440 (69.0)		0.91 (0.73, 1.12)	0.85 (0.68, 1.08)
≥3	41.23 (9.04)		421 (62.0)		0.67 (0.54, 0.82)	0.67 (0.53, 0.84)
The time of previous screening						
Over 3 years	41.22 (9.05)	0.001	351 (61.5)	<0.001	1.00 (reference)	1.00 (reference)
Within 3 years	42.62 (8.57)		1231 (69.9)		1.46 (1.20, 1.77)	1.58 (1.27, 1.97)
The result of previous screening						
Normal	41.91 (8.78)	<0.001	1154 (65.6)	<0.001	1.00 (reference)	1.00 (reference)
Abnormal	42.08 (9.03)		85 (72.0)		1.35 (0.89, 2.04)	1.24 (0.80, 1.92)
Unknown	43.75 (8.21)		343 (75.4)		1.61 (1.27, 2.03)	1.42 (1.11, 1.82)

a P for t test or one-way ANOVA.

b P for chi-square test.

c Variables with P ≤0.10 in the uni-variate analysis were included in the multi-variate logistic regression model. Bold values indicate statistical significance (P < 0.05).

### Factors Associated With Pre-Procedural Anxiety Among Women Without PSE

Factors associated with pre-procedural anxiety among women without PSE were found in the multi-variate logistic regression model (**Table 2**). Higher odds of being anxious were shown if women were older (OR: 1.57, 95%CI: 1.15~2.15), married (OR: 2.33, 95%CI: 1.37~3.97), nonparous (OR: 2.38, 95%CI: 1.01~5.61), and having psychological distress (OR: 2.85, 95%CI: 1.94~4.19). Potential protective factors of anxiety included receiving higher education (OR: 0.45, 95%CI: 0.31~0.65), having older age at first delivery (OR: 0.50, 95%CI: 0.28~0.91), walking more steps per day (OR: 0.63, 95%CI: 0.48~0.82), and having heard of cervical cancer screening (OR: 0.37, 95%CI: 0.25~0.56).

### Factors Associated With Pre-Procedural Anxiety Among Women With PSE

Distinct associated factors were detected among women with PSE (**Table 3**). Women who were susceptible to pre-procedural anxiety were identified as: having two or more sexual partners (OR: 2.11, 95%CI: 1.13~3.93), sitting longer per day (OR: 1.25, 95%CI: 1.03~1.51), having psychological distress(OR: 3.00, 95%CI: 2.30~3.91), receiving screening within three years (OR: 1.58, 95%CI: 1.27~1.97), and unknowing previous screening results (OR: 1.42, 95%CI: 1.11~1.82). Women that were less likely to be anxious tended to receive higher education (senior middle school: OR: 0.61, 95%CI: 0.49~0.77; college or above: OR: 0.59, 95%CI: 0.45~0.77), earn higher monthly income (OR: 0.79, 95%CI: 0.64~0.92), be diagnosed with vaginitis (OR: 0.77, 95%CI: 0.64~0.92), do physical exercise per week (two times: OR: 0.66, 95%CI: 0.51~0.86; three or more times: OR: 0.63, 95%CI: 0.48~0.83), and participating three or more times screening (OR: 0.67, 95%CI: 0.53~0.84).

## Discussion

Negative psychological response to cervical cancer screening procedures has been considered to be a barrier to screening uptake. The present study explicitly investigated the prevalence of pre-procedural anxiety during cervical cancer screening among Chinese females using a cross-sectional design. Overall, nearly three quarters of the participants suffered pre-procedural anxiety, suggesting the substantial psychological harm derived by cervical cancer screening. To our knowledge, this study is a forerunner to explore the influence of PSE on the anxious symptom prior to the cervical cancer screening procedures. Notably, PSE may bring varied effects on the pre-procedural anxiety due to the difference of past screening times, interval and results. These novel findings help to develop proper guidance in reducing the psychological harm and promoting more uptake of cervical cancer screening.

Scant studies investigate the pre-procedural anxiety symptom in cervical cancer screening. This study reported a high level of the pre-procedural anxiety, with over 70% women rating a score above 40 in the STAI-S scale. The mean score (42.72) was much higher than that (30.2) in the USA (10). A relatively lower mean score (33.0) has also been detected before women underwent a Pap smear in the Netherlands (18). Despite of the ethnic, culture, and socio-economic differences, this disparity may also result from the distinct knowledge of HPV and cervical cancer. It has been revealed that better HPV knowledge was associated with lower anxiety and concerns during screening (19). Therefore, the prevalent pre-procedural anxiety in our survey may be partly explained by the knowledge gaps about HPV between China and other developed countries that we have previously found (20). Interestingly, the anxious level before screening was likely to be weaker than that in the diagnosis stage. Irish researchers observed a higher mean score of the STAI-S scale (45.31) prior to colposcopy (21). Colposcopy is usually applied for further diagnostic evaluation after receiving abnormal cervical cancer screening results. The fear of being diagnosed with malignancy along with complicated operations may bring more worries during colposcopy than screening procedures. Pre-procedural anxiety may associate with colposcopy-related pain and discomfort (22, 23). Nevertheless, our findings support the urgent need for the delivery of psychological assessment and support to the female population before screening procedures start.

Past participation of cervical cancer screening may help to reduce the anxiety or other negative psychological reactions in the current screening round. In our study, women without PSE had higher level of pre-procedural anxiety than their experienced counterparts. Similar findings have also been observed in other types of cancer screening. For colorectal cancer screening, patients without previous experience demonstrated greater anxiety when undergoing colonoscopy (24). For breast cancer screening, women who received mammogram at the first time tended to be more distressed than those having prior mammograms (25). However, the impact of PSE on screening-related anxiety may be obscured by a family history of cancer diagnosis. There was a inconsistent finding among women with a family history of breast cancer that women who had undergone mammography screening previously were vulnerable to longer-term distress (26). The possible explanation lies that a woman is more stressful to receive screening services regardless of having PSE if her relative has been diagnosed with or died from cancer. In addition, we noticed that the prevalence of pre-procedural anxiety decreased when the times of past screening participation increased in our study. This contrasted with another study in breast cancer screening, in which the anxiety level increased with the number of previous mammograms done (27). The difference in screening methods, medical apparatus and instruments, and body parts lead to these inconclusive findings to some extent, however, other screening-related factors may play a potential role in the link between PSE and anxiety, such as screening frequency, and past screening results.

In our study, distinct associated factors of pre-procedural anxiety between women with and without PSE were found, especially variables specific to PSE. Among women with PSE, we confirmed the impact of past screening times on pre-procedural anxiety that women participating three or more times screening had less likelihood of being anxious. A similar result were detected among women without PSE that having heard of cervical cancer screening was associated with less anxiety. Both more screening participation and heard of screening indicate a better understanding of the screening procedures, which may help women to reduce the psychological discomfort in cervical cancer screening. Notably, this protective effect may be counteracted by a short screening interval (within three years) and uncertainty of previous screening results as we observed in the present study. According to the screening guidelines, women can be screened every three or five years unless positive screening results for HPV testing or cytology appear (28). This means that screening repeatedly within three years is more likely to be owing to abnormal screening results, which may bring a heavier psychological burden (4, 29, 30). Furthermore, overscreening may present in these women, which can also result in significant anxiety (31). For women unknowing past screening results, less self-confidence and more worries in health status may become more salient when they engage in a new round of screening. Consedine et al. has proposed that there are different sources of anxiety in cancer screening, including fear of the screening process (e.g. pain, discomfort, embarrassment), fear of the screening outcomes, and undifferentiated fear of cancer (32). Hence, it can be inferred that PSE affects the sources of pre-procedural anxiety in different manners. Fear of the screening process may be alleviated by past participation of screening, while fear of the screening outcome or getting cancer may be aggravated by a short screening interval and previous uncertain results. Further population-based investigations are needed to verify the contribution of PSE to pre-procedural anxiety with different sources in cervical cancer screening.

Study limitations were shown in the present study. As the study sample came from only one screening center as well as smartphone users, the prevalence of pre-procedural anxiety might be not able to generalize to the whole population in cervical cancer screening. Recall bias on PSE and other key information could not be avoided due to self-reported answers. Moreover, we conducted psychological assessment prior to the screening process rather than during the screening procedure, which might lead to underestimation of anxious feeling. The sources of anxiety could not be distinguished in our study as well. Thus, precise classification evaluation of screening related anxiety should be considered in further investigations. In addition, Women’s psychological health status may be influenced by the COVID-19 pandemic to some extent. However, there was no difference of psychological distress in recent two weeks between women with and without PSE. Simultaneously, no local COVID-19 cases had been detected in Shenzhen during the survey time period. The impacts of the COVID-19 pandemic could be limited.

In conclusion, the current study lends to support that women participating in cervical cancer screening commonly present pre-procedural anxiety. Importantly, PSE may help to alleviate pre-procedural anxiety, which is influenced by past screening times, interval, and results. Even if women have not been screened before, having heard of cervical cancer screening is associated a lower likelihood of pre-procedural anxiety. Psychological counseling according to women’s PSE before cervical cancer screening is warranted of necessity.

## Data Availability Statement

The raw data supporting the conclusions of this article will be made available by the authors, upon reasonable request.

## Ethics Statement

The studies involving human participants were reviewed and approved by the medical ethics committee of Shenzhen Maternity and Child Healthcare Hospital. The patients/participants provided their written informed consent to participate in this study.

## Author Contributions

WL wrote and presented the original draft. WL, WH, CZ, and LZ were involved in data curation and visualization. WL, WH, CM, PL, ZL, and YW were involved in methodology, software, analysis, review and editing. WL, WH, and CM revised the manuscript and polished the language. SY and YW were involved in supervision. All authors contributed to the article and approved the submitted version.

## Funding

This study was supported by the Shenzhen Healthcare Research Project (Grant No. SZGW2018005) and the Sanming Project of Medicine in Shenzhen (Grant No. SZSM201612042).

## Conflict of Interest

The authors declare that the research was conducted in the absence of any commercial or financial relationships that could be construed as a potential conflict of interest.

## Publisher’s Note

All claims expressed in this article are solely those of the authors and do not necessarily represent those of their affiliated organizations, or those of the publisher, the editors and the reviewers. Any product that may be evaluated in this article, or claim that may be made by its manufacturer, is not guaranteed or endorsed by the publisher.
